# Whole-Genome Sequencing Identified a Novel Mutation in the N-Terminal Domain of *KIF5A* in Chinese Patients with Familial Amyotrophic Lateral Sclerosis

**DOI:** 10.3390/genes15060680

**Published:** 2024-05-24

**Authors:** Hui Wang, Liping Guan, Xiaojuan Ma, Yiying Wang, Jinhao Wang, Peipei Zhang, Min Deng

**Affiliations:** 1 Institute of Medical Innovation and Research, Peking University Third Hospital, Beijing 100191, China2010301154@stu.pku.edu.cn (J.W.); 2 Laboratory of Genomics and Molecular Biomedicine, Department of Biology, University of Copenhagen, 1550 Copenhagen, Denmark; 3 Department of Biochemistry and Molecular Biology, School of Basic Medical Sciences, Peking University Health Science Center, Beijing 100191, China

**Keywords:** amyotrophic lateral sclerosis, genetics, *KIF5A*, clinical neurology, neurogenetics

## Abstract

Amyotrophic lateral sclerosis (ALS) is a devastating neurodegenerative disorder characterized by progressive damage to both upper and lower motor neurons. Genetic factors are known to play a crucial role in ALS, as genetic studies not only advance our comprehension of disease mechanisms but also help unravel the complex phenotypes exhibited by patients. To gain further insights into the genetic landscape of ALS in the Chinese population and explore genotype–phenotype correlations among individuals, we conducted whole-genome sequencing to screen genes in 34 Chinese familial ALS (FALS) probands lacking the most common ALS-associated genes. Within this cohort, we identified a rare heterozygous missense mutation in the N-terminal domain of *KIF5A* (c.86A>G) in one of the probands. This finding is significant as mutations in the *KIF5A* gene have been implicated in ALS in European cohorts since 2018, predominantly characterized by C-terminal mutations. Analysis of the clinical phenotype within this familial lineage revealed a delayed onset of symptoms, an extended survival duration, and initial manifestations in both upper limbs. These observations underscore the clinical heterogeneity observed in ALS patients harboring *KIF5A* mutations. In conclusion, our study contributes to the growing body of evidence linking *KIF5A* to ALS and enhances our understanding of the intricate genetic landscape of this disease.

## 1. Introduction

Amyotrophic lateral sclerosis (ALS) is a fatal neurodegenerative disease that progressively damages both upper and lower motor neurons. As ALS advances, it leads to the gradual degeneration of motor neurons, muscular atrophy, functional impairment, and, eventually, paralysis. Patients typically succumb within three to five years following symptom onset, with respiratory failure as the primary cause of death [1]. ALS is classified into familial ALS (FALS) and sporadic ALS (SALS). FALS accounts for approximately 10% of all ALS cases, and the other cases are categorized as sporadic amyotrophic lateral sclerosis (SALS) [2]. Understanding the etiology of ALS is crucial for developing personalized treatment strategies.

Among numerous factors, genetics plays an important role in ALS pathogenesis, with over 40 ALS-related genes identified [3]. Many new ALS-related genes and the complex genetic mechanisms of ALS, such as oligogenic inheritance, are also under investigation. In 2018, Nicolas, A. et al. [4] identified kinesin family member 5A (*KIF5A*) as a novel gene associated with ALS by comparing genome-wide association study (GWAS) data from a total of 20,806 ALS patients and 59,804 controls. Similarly, in the same year, Brenner et al. conducted a comparative analysis of whole-exome sequencing (WES) data from 426 FALS patients and 6137 controls, confirming *KIF5A* as an ALS-associated gene [5]. These studies revealed that ALS-associated mutations in *KIF5A* were primarily located in the structural domain of the C-terminal tail, and the patients exhibited the classic phenotype of ALS. The classic phenotype of ALS involves simultaneous upper motor neuron and lower motor neuron signs, presenting with weakness starting in the limbs [4,5].

*KIF5A* is a member of the dynamin proteins family, which is composed of a globular motor domain located in the head, an α-helix coiled-coil stalk domain, and a globular domain situated in the tail [6]. The N-terminus region of *KIF5A* contains an ATP-binding sequence and a microtubule-binding sequence that regulates the movement of microtubules. The stalk domain is responsible for interacting with other subunits and can bind to kinesin light chains associated with specific cargos. The tail can directly bind to both cargos and kinesin light chains [7]. *KIF5A* plays a pivotal role in facilitating the transportation of granules that carry RNA and RNA-binding proteins between neuronal dendrites and axons. Besides ALS, mutations in *KIF5A* are associated with other motor neuron diseases. Interestingly, different diseases appear to be associated with specific mutation sites. For instance, mutations in the N-terminus region of the gene are prevalent causes of autosomal dominant hereditary spastic paraplegia (HSP) in European populations, as well as the genetic etiology of Charcot–Marie–Tooth type 2 (CMT2) [8,9]. These mutations disrupt protein and RNA interactions, which subsequently leads to the dysregulation of gene expression, changes in RNA splicing, disruptions in axonal transport, and a decrease in neuronal survival [10]. However, as mentioned above, mutations specifically situated in the C-terminal hotspot region of the *KIF5A* gene lead to a distinct manifestation of ALS [5]. Due to the genetic overlap among these motor neuron diseases and the partial similarity in clinical phenotypes, it is worth investigating whether mutations in the N-terminal region of *KIF5A* are associated with ALS.

The genetic landscape of ALS varies among populations, with distinct genetic compositions contributing to different disease characteristics. Variations in the genetic composition contribute to distinct characteristics of ALS across different ethnicities, with a notable prevalence of cases lacking identifiable pathogenic mutations among Chinese individuals. Clinically, Chinese patients often experience an earlier age of onset, a lower incidence of bulbar-onset ALS, and generally more favorable prognoses. The most prevalent mutation in European cases was C9orf72, while the most common mutation in Asian cases was SOD1 [11,12,13,14]. Currently, given the notable disparity in genetic epidemiology among different nations [12,15] and the lack of large-scale genetic screening studies in Chinese ALS patients, it is important to conduct comprehensive genetic screening to elucidate the unique genetic structure of ALS in China. Additionally, further research is needed to investigate the spectrum of *KIF5A* mutations and related phenotypic characteristics in the Chinese ALS population. Therefore, our study recruited 127 Chinese ALS probands and conducted comprehensive whole-genome sequencing on 34 of them to analyze potential pathogenic mutations, and we specifically focused on *KIF5A* mutations and associated phenotypic characteristics.

## 2. Material and Methods

### 2.1. Study Population

The study recruited a cohort of 127 individuals diagnosed with FALS who were admitted to the Department of Neurology at Peking University Third Hospital between 2003 and 2018. Positive family history was considered if the patient had at least one affected relative within two generations. All the patients with ALS were diagnosed based on the El Escorial revised criteria for definite or probable ALS [16]. Hand-signed informed consent was obtained from each participant. The project has passed the ethics review of Peking University Third Hospital (No. IRB00006761-L2010055). Demographic and clinical information of all patients was collected (Table 1). Additionally, regular telephonic follow-ups were conducted with the patients. Disease severity was assessed using the ALS Function Rating Scale (ALSFRS).

### 2.2. Sample

The genomic DNA was extracted from the peripheral blood of the patients using the QIAamp^®^ DNA Blood Mini Kit (Qiagen, Valencia, CA, USA) following the instructions provided by the manufacturer and quantified by a Qubit 3.0 fluorometer (Life Technologies, Paisley, UK). We pre-screened the 127 FALS probands for common variants in the four most prevalent ALS causative genes (*SOD1*, *FUS*, *TARDBP*, and *C9orf72*) using fragment-length and repeat-primed PCR, followed by Sanger sequencing. Families lacking disease-causing mutations in these four genes were deemed to harbor other less common genetic mutations. From these families, we selected 34 probands from 34 pedigrees to perform whole-genome sequencing (WGS) to identify the likely pathogenic mutation(s) that contributed to the occurrence of FALS.

### 2.3. Bioinformatic Analysis

Whole-genome sequencing (WGS) was employed to detect genetic variants, including single nucleotide variants (SNVs), short insertions-deletions (INDELs), short tandem repeats (STRs), copy number variants (CNVs), and structure variants (SVs). The BGISEQ-500 platform, which utilizes DNA nanoball and probe-anchor synthesis technologies, was employed for DNA library construction, quality control, pooling, and sequencing. Each sample yields an average of 1354 million 100 bp pair-end reads. Raw sequencing reads that contained a fraction of gaps (N) or low-quality bases (Q < 20) at a threshold of >0.1 were removed using SOAPnuke [17] (version 2.0.1). The percentage of clean reads with a base quality > 20 is approximately 99%. The clean reads were aligned to the human reference genome (hg38) using the Burrows–Wheeler aligner [18] (version 0.7.15) with default settings. The aligned reads were stored in cram format, and the alignment summaries were calculated by samtools [19] (version 1.15.1). These summaries included the average sequencing depth, average coverage, and average map rate (Supplementary Table S1).

SNVs and INDELs were detected by the Genome Analysis Tool Kit [20] (version 3.6). Variants were excluded based on the following quality control (QC) criteria: (1) read depth < 4, (2) map quality < 55, (3) variant quality < 30, and (4) genotype quality < 30. The minor allele frequency (MAF) of the variants was accessed using the Genome Aggregation Database (gnomAD) [21] (version 3.1). Repeat-expansion calling was performed with expansionHunter [22] (version 5.0.0), and variants with a quality < 30 or coverage depth < 10× were excluded. CNV calling was detected using CNVnator [23] (version 0.4) with a bin size of 100 bp. The coordinates of all samples’ CNVs were merged together using bedtools [24] (version 2.30.0), with a 1 bp overlap. CNVs were excluded based on QC criteria recommended by the author: (1) Q-value > 0.05, (2) fraction of reads with zero map quality > 0.5, and (3) fraction of gaps > 0, and (4) size < 1000 bp or > 100,000 bp.

SVs calling was performed by LUMPY [25] (version 0.2.13) and genotyped using SVTyper [26] (version 0.1.4). A bed file (https://github.com/hall-lab/speedseq/blob/master/annotations/exclude.cnvnator_100bp.GRCh38.20170403.bed (accessed on 12 April 2024)) marked the complex region of human genome was used for excluding calling. The copy numbers of SVs, including deletion and duplication, were calculated by CNVnator at the SV coordinates. A part of duplication and deletion SVs were reclassified into breakend and/or MEI according to the copy number and repeat mask in the hg38 genome. We employed the SV filtering criteria from Abel’s study [27]. SVs with any of the following quality control criticisms were excluded: (1) the proportion of split-read and paired-end read counts < 10%, (2) the mean sample quality < 150, (3) the deletion size < the insert size of sequencing library estimated by SVTyper, and (4) deletion copy number estimated by CNVnator [23] > 0.5 or duplication copy number < 1.5.

The Variant Effect Predictor (VEP, version 107, [28]) was employed to provide comprehensive annotations for SNPs and small INDELs based on the VCF file. These annotations encompassed essential details such as the gene name, transcript, mutation consequence, and allele frequency, which were sourced from public databases, including the Genome Aggregation Database (gnomAD), the 1000 Genome Project, and the Exome Aggregation Consortium (ExAC)). Variants with population frequencies exceeding 0.005 in the public database (gnomAD, version 3.1) were excluded. By incorporating the interpretation information from the ClinVar database into these reserved variants, the variants identified in this study were categorized according to the standards and guidelines of the American College of Medical Genetics and Genomics (ACMG) as pathogenic (P), likely pathogenic (LP), uncertain significance (VUS), likely benign (LB), benign (B), or conflicting, in cases where there was a disagreement between ClinVar and Varsome [29] in their assessment. The Annotation and Ranking of Human Structural Variations (AnnotSV) [30] was used to provide comprehensive annotations for SV, CNV, and STR. AnnotSV assessed the pathogenicity of the variants and provided scores and classifications ranging from class 5 to class 1, which correspond to P, LP, VUS, LB, and B, respectively.

### 2.4. Mutation Screening

To further investigate, we collected and systematically listed all the genes related to ALS through OMIM (An Online Catalog of Human Genes and Genetic Disorders, https://www.omim.org/), PubMed (PubMed (nih.gov)), Google Scholar (https://scholar.google.com/), MGI database (MGI—Mouse Genome Informatics—The international database resource for the laboratory mouse; https://www.informatics.jax.org/) and ALSoD (Amyotrophic Lateral Sclerosis online Database, ALSoD, https://alsod.ac.uk/). The total number of genes was 114. Upon the knowledge, these genes were divided into eight categories: causative gene (29), loci-associated gene (2), MGI-associated gene (7), susceptibility to ALS gene (10), associated gene (13, including modified gene (8), associated FUSion gene (1), and associated therapy gene (3)), and ALSoD gene (46), including strong evidence gene (6), moderate evidence gene (7), tenuous evidence gene (33), conflicting gene (3), and weakness evidence gene (5). All 114 genes are listed in Supplementary Table S2.

We applied stringent criteria to filter SNPs and small INDELs from WGS data. Firstly, we retained variants located in the coding region of 114 ALS-associated genes. Additionally, we preserved variants predicted to impact protein structure and function based on their consequence types, which include splice_acceptor_variant, splice_donor_variant, stop_gained, frameshift_variant, stop_lost, start_lost, inframe_insertion, inframe_deletion, missesnse_variant, and TFBS_amplification. Post-filtering, each sample yields a reduced number of variants. For causative genes, we classified variants according to ACMG into P/LP/VUS/LB/B. For non-causal genes, we retained harmful variants that were marked by their rarity and specific types known to affect protein function. The VEP (version 107) software has numerous plugins for predicting the pathogenicity of variants. We have referred to several mainstream predictive software tools, such as REVEL (version 1.3), CADD (version 1.5), Condel (version 1.0) (based on the pre-calculated SIFT and PolyPhen-2), and SpliceAI (version 1.3). Under equivalent conditions, variants predicted to be harmful by multiple software or tools will be given priority. Regarding CNVs, SVs, and STRs, we preserved variants that met the following two conditions: those classified as pathogenic or likely pathogenic by AnnotSV and those where the variant’s genomic region overlapped with any of the 114 ALS-associated genes.

## 3. Results

### 3.1. Mutation Analysis

**Figure 1:**
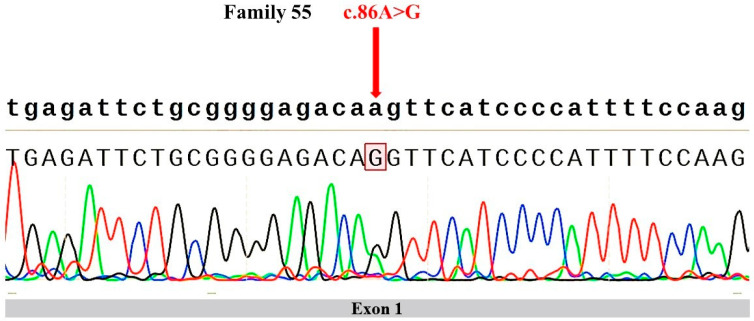
ALS-associated mutations identified in *KIF5A.* The colors represent the different nucleotides: Green for Adenine (A), Red for Thymine (T), Blue for Cytosine (C), and Black for Guanine (G).

Among the cohort of 127 FALS probands, only a single rare heterozygous missense mutation (p.K29R) was identified in the *KIF5A* in the proband of Family 55 (F55) (Table 2). The same mutation was found in the patient’s son, who did not show symptoms of ALS at the age of 35. The presence of the variation was confirmed using Sanger sequencing (Figure 1). There was no record of this variation in the gnomAD v3 and the ExAC database. In gnomAD v4.0.0, this variant is present in two East Asian individuals; the mutation frequency is 0.00005038. No pathogenic variants in other candidate genes were found in the proband. Multiple tools were employed to predict the pathogenicity of this mutation (Table 3). Both CADD and M-CAP models indicated that this mutation is likely to be pathogenic. Furthermore, we used PhyloP100way to assess the conservation of this mutation. PhyloP100way calculates conservation scores for each site based on multiple sequence alignments of 100 vertebrate species. The mutation scored 5.85, indicating a high level of conservation.

### 3.2. Clinical Information

**Figure 2:**
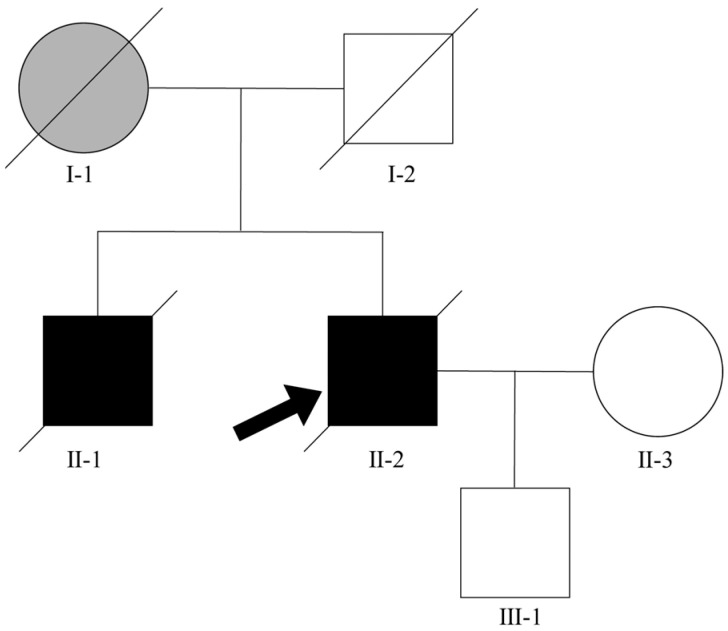
Diagram of Family 55.

The proband of Family 55 harboring the exon 1 heterozygous missense mutation c.86A>G in *KIF5A* was a Han male from mainland China. The family tree for this pedigree was illustrated (Figure 2). At the age of 60, the patient began experiencing weakness in both upper limbs and an impaired ability to elevate them. The condition progressively worsened, with visible muscle atrophy in the upper limbs. One year later, weakness in the lower limbs emerged, along with unsteady walking. After a period of 51 months from the symptom onset, then he came to our outpatient clinic to seek medical attention. The neurological examination revealed that muscle strength of his biceps brachii, triceps brachii, and the deltoid muscle in both upper limbs were graded as Ⅲ+ (movement against gravity and minor resistance applied by the examiner is possible), Ⅱ (movement at the joint is possible, but only when gravity is eliminated), and Ⅲ (movement against gravity is possible, but not against resistance applied by the examiner), respectively. Both lower limbs have abnormal muscle strength, with the anterior tibialis and gastrocnemius muscles displaying a grade of Ⅴ (normal muscle strength, where the muscle can move the joint against full resistance) and Ⅴ- (muscles can do most of the range of motion of the joint against resistance), respectively. The bilateral reflexes of the biceps, triceps, knee, and ankle were observed to be within normal parameters. The results of the bilateral Babinski and Hoffmann tests yielded negative findings. The patient’s Ⅻ cranial nerve (the hypoglossal nerve) was damaged, resulting in fasciculation of the tongue muscles. Muscle atrophy is observed in all four limbs, with a predominant involvement of the proximal musculature. The patient had normal cognitive function and possessed a familial background that indicated a predisposition for ALS. His sibling similarly exhibited signs of muscle atrophy and weakness approximately 5 months before his own diagnosis.

We also tried to perform a segregation analysis on the proband’s family. The identical mutation was identified in his son, who had not yet manifested any symptoms of ALS during the aforementioned period. Regrettably, the blood sample of his brother, who was also diagnosed with ALS, was not obtained for the purpose of genetic screening.

The shape of the empty circle means asymptomatic female, and the solid circle means female patient. The shape of the empty square means asymptomatic male, and the solid means male patient. The shape of the gray circle means female with neurological disorders. The slash crossing circle or square means death. The arrow directs the proband of pedigree. The horizontal line describes the marital relationship, and the vertical line describes the parent–child relationship.

## 4. Discussions

In this study, we performed genetic screening on 127 probands with FALS, 34 of them undergoing WGS. We elucidate the impact of these variants on the prevalence and clinical features in the cohort of FALS. Among them, we identified a *KIF5A* p.K29R mutation in one FALS patient. This missense mutation leads to an alteration in the amino acid composition from lysine(K) to arginine(R) at position 29. Notably, this is the first time that mutations in the N-terminal domain of the *KIF5A* gene have been directly linked to ALS, thus expanding the spectrum of mutations implicated in the pathogenesis of ALS. This discovery lays the foundation for a comprehensive exploration of the disease’s pathogenic mechanisms.

*KIF5A* is a member of the dynamin proteins family, which plays a pivotal role in facilitating the transportation of granules that carry RNA and RNA-binding proteins between neuronal dendrites and axons. Notably, this cargo comprises essential proteins associated with ALS, such as FUS and hnRNPA1 [5]. Moreover, *KIF5A* functions in facilitating the trafficking of vasoactive amine-binding protein (VABP) and plays a role in facilitating the axonal transport of neurofilaments. It has been observed that the absence of *KIF5A* in mice leads to atypical *KIF5A* neurofilament transport [31]. Recent studies have revealed that *KIF5A* is also expressed in astrocytes of SOD1 mutant ALS patients, and improving *KIF5A*-dependent transport may improve astrocyte process-mediated support of neuronal networks [32].

Mutations in the *KIF5A* gene have been linked to various disorders, such as hereditary spastic paraplegia (HSP), Charcot–Marie–Tooth type 2 (CMT2), and ALS. Previous studies have indicated that missense mutations occurring in the N-terminal motor domain of *KIF5A* are associated with HSP and CMT2 [8,9,33]. Conversely, mutations affecting the splicing of exon 27 in the C-terminal region have been found to be causative to ALS. *KIF5A* mutations are considered a susceptibility factor for ALS [4,5]. *KIF5A* mutations have been reported to account for 0.16–0.41% of sporadic ALS patients within the Chinese population [34,35,36]. In a separate study conducted by Naruse H et al., WES analysis was conducted on a cohort of Japanese patients. The results revealed the presence of loss-of-function variants exclusively in two FALS. Their clinical features of the condition encompass the presence of upper motor neuron impairment [37]. In another study by Zhang Xue et al., *KIF5A* sequences were analyzed in a large Chinese ALS cohort, where the prevalence of *KIF5A* mutations among SALS in China accounted for 0.16% (1/645) [34]. In ALS, mutations linked to *KIF5A* typically follow an autosomal dominant inheritance pattern. Previous research has shown that patients with *KIF5A* mutations often exhibit prominent symptoms in a heterozygous state, indicating a dominant mode of inheritance for these mutations. For instance, Nicolas et al. [4] reported six heterozygous loss-of-function variants in the *KIF5A* gene among FALS patients. Similarly, R. Nakamura et al. identified seven different *KIF5A* mutations in 13 out of 807 Japanese SALS patients, all of which were heterozygous [38]. This suggests that the majority of *KIF5A* heterozygous mutations demonstrate dominant characteristics. In this study, we only identified a heterozygous missense mutation in the *KIF5A* gene in one of the probands. Together with previous reports from China, this finding further illustrates that mutations in the *KIF5A* gene are rare among individuals of Chinese descent.

The missense mutation we detected was located on exon 1 of the N-terminal domain of the *KIF5A* gene (p.K29R). As per the standards and guidelines of the American College of Medical Genetics and Genomics (ACMG), this variant is classified as having unknown significance (VUS). Previous research has found that mutations associated with ALS are located within the intronic regions of the *KIF5A* gene. These mutations induce incorrect splicing of *KIF5A* messenger RNA (mRNA), thereby causing the mis-splicing of exon 27 [4,5]. Hence, certain investigations propose that the impairment of *KIF5A* functionality serves as a contributing element in the development of ALS. Nevertheless, subsequent studies have revealed that pathogenic mutations associated with ALS lead to a toxic gain of function in the intracellular motor protein *KIF5A*, disrupting intracellular transport and neuronal homeostasis [10]. In vitro studies have revealed that mutations in *KIF5A* associated with ALS have a propensity to cluster, display heightened microtubule motions, and possess neurotoxic properties *KIF5A* [39,40]. The aforementioned studies cumulatively indicate that ALS-associated *KIF5A* mutations exhibit a deleterious gain of function.

Our research raises questions about the detection of ALS-related mutations in the N-terminal domain of *KIF5A*, diverging from the prevailing notion of mutations predominantly in the C-terminal domain. Missense mutations occurring in the N-terminal domain have an impact on microtubule binding and/or ATP hydrolysis. Consequently, this results in impairments in the transportation of cargo mediated by *KIF5A* in dendrites and axons [41]. These findings align with the pathogenic mechanisms observed in the aforementioned C-terminal mutations. These findings suggest a shared mechanism underlying the pathogenic effects of both C-terminal and N-terminal variations in *KIF5A*.

Regarding the relationship between genotype and phenotype, Brenner et al. have documented that patients with the *KIF5A* loss-of-function variant who suffer from ALS display clinical characteristics that are in line with classical ALS [4]. Conversely, Nicolas et al. have reported that ALS patients carrying the *KIF5A* loss-of-function variant exhibit an earlier age of onset and a prolonged survival period [5]. In a recent case report, a patient displayed features overlapping ALS, HSP, CMT2, and frontotemporal dementia and had a mild disease course with prolonged survival. However, the rate of progression and survival time differed among family members, suggesting that ALS patients with mutations in this gene have great clinical heterogeneity [42]. In 2019, Fan Dongsheng et al. reported the identification of a c.2999delC mutation in China. This mutation was observed in a cohort of 581 patients with SALS, while a control group of 1015 individuals without a history of neurological disease did not exhibit this mutation. The c.2999delC mutation was shown to result in the deletion of a stop codon and subsequent elongation of the protein [36]. In our research, the onset age of patients in Family 55 in our cohort was 60 years, which is consistent with a prior study that found the median age at onset for Chinese ALS patients to be 52 ± 12 years [43,44]. After a period of 18 months of post-onset monitoring, it was observed that the ALSFRS score and body weight of this patient exhibited no significant changes. The patient died 68 months after the onset of symptoms, longer than the reported median survival of 33 months for patients in China [44]. Therefore, in comparison to previously reported Chinese patients with *KIF5A* mutations, this patient exhibited a delayed onset of symptoms, an extended time of survival, and initial symptoms appearing in both upper limbs. These findings suggest that there is variability in the clinical features observed in ALS patients with mutations in the *KIF5A* gene.

Our study has several limitations. Firstly, due to the limited number of patient samples, there may be an impact on the statistical power to detect rare variants, leading to sampling bias in the analysis. Moreover, our segregation analysis of this pedigree lacks comprehensiveness, preventing us from elucidating the specific role of this variant within the family. Secondly, public databases like gnomAD contain vast genomic data, but their sample sources and coverage may have limitations. Especially for specific populations like the Chinese, genetic variations may differ significantly from other populations. Therefore, solely relying on public databases for variant filtering may not fully reflect the genetic characteristics of the studied population, potentially introducing population bias. Hence, it is better to cautiously consider the use of public databases in variant analysis and strive to validate results with additional data sources to minimize potential biases. Additionally, we lacked cellular-level experiments to validate the precise effects of this mutation on *KIF5A*. In the future, we plan to utilize patient skin fibroblasts for induced differentiation and further investigate the pathogenicity and pathogenic mechanism of this mutation using cell models or mouse models. Indeed, although short-read sequencing technologies are widely used in genomic sequencing due to their high throughput and cost-effectiveness, they do have certain limitations when it comes to detecting structural variations (SVs). Short-read limits their ability to resolve small insertions or deletions, especially when the size of these variations is smaller than the read length. For larger structural variations, such as chromosomal rearrangements, inversions, or large insertions/deletions, short-read technologies struggle to accurately identify the breakpoints, as these events often span across multiple reads. In some cases, complex structural variations that involve multiple breakage and rejoining events can be particularly challenging to resolve using short-read sequencing technologies. Due to the above limitations, short-read technologies may have a higher rate of false positives or false negatives when detecting SVs.

In this study, we performed whole-genome sequencing in a cohort of Chinese FALS patients. Among them, one patient was found to carry a novel pathogenic N-terminal heterozygous missense variant, p.K29R, in the *KIF5A* gene. The patient’s phenotype, characterized by delayed onset, prolonged survival, and initial upper limb symptoms, further illustrates the heterogeneity of phenotypes among ALS patients with *KIF5A* mutations. This sequential research on specific individuals will contribute to our comprehensive understanding of the disease process, thereby establishing a fundamental basis for disease identification and management. Further studies are needed to elucidate the effect of genetic variants and the precise function of the *KIF5A* gene in ALS.

## Figures and Tables

**Table 1:** Demographic features of patients with ALS in the study.

	All Patients (n = 127)	Patient Carrying *KIF5A* p.K29R Variant and CNV
Age, years	45.5 ± 12.45 ^a^	64
Sex, num (%)		
- Male - Female	80 (63%) 47 (27%)	Male
Age at onset, years	41.86 ± 13.25 ^a^	60
Site of onset, num (%)		
- Bulbar - Spinal - Unknown	15 (11.8%) 111 (87.4%) 1 (0.8%)	Spinal
Diagnosis delay, months	29 (13.0–69.5) ^b^	51
Survival time, months	54 (26–89) ^b^	68
ALSFRS score at diagnosis	33.47 ± 6.75 ^a^	31

SD, standard deviation; IQR, interquartile range; F, female; M, male; ALSFRS, Amyotrophic Lateral Sclerosis Function Rating Scale; CNV, copy number variation. ^a^ mean ± SD; ^b^ median (IQR); the “Age” in the table was the age when the patient first visited Peking University Third Hospital.

**Table 2:** Description of mutations identified in Family 55.

Gene	Mutation	cDNA	dbSNP	Coordinates	ExAC (EAS)	gnomAD v3
*KIF5A*	p.K29R	c.86A>G	-	12:57550357	-	-

Genomic coordinates are based on Genome Reference Consortium Human Build 37 (GRCh37/hg19). ALS, amyotrophic lateral sclerosis; cDNA, complementary DNA; dbSNP, The Single Nucleotide Polymorphism Database; ExAC (EAS), The Exome Aggregation Consortium (East Asia).

**Table 3:** Pathogenicity prediction for *KIF5A* p.K29R variant.

Scheme 100	PolyPhen	MutationTaster	FATHMM	M-CAP	REVEL	CADD	PhyloP100way	phastCons100way
Tolerated (0.1)	Benign (0.012)	NA	T	D	0.243	23.3	5.85	1

SIFT, Sorting Intolerant from Tolerant; PPH2, Polymorphism Phenotyping v2; CADD, Combined Annotation-Dependent Depletion; FATHMM, Functional Analysis through Hidden Markov Models; M-CAP, Mendelian Clinically Applicable Pathogenicity; REVEL, Rare Exome Variant Ensemble Learner; T, tolerated; D, deleterious.

## Data Availability

No new data were created or analyzed in this study. Data sharing is not applicable to this article.

## Supplementary Materials

The following supporting information can be downloaded at https://www.mdpi.com/article/10.3390/genes15060680/s1.
