# Supplementary material for: Whole-Genome Sequencing Identified a Novel Mutation in the N-Terminal Domain of KIF5A in Chinese Patients with Familial Amyotrophic Lateral Sclerosis
Source: Genes (Basel). 2024 May 24;15(6):680. doi: 10.3390/genes15060680 (PMC11203265; doi:10.3390/genes15060680)
Supplement: Supplementary file 1 [file genes-15-00680-s001.zip › genes-2986974-supplementary.pdf]

Supplementary Table S1. Alignment statistics

Summary	Mean±sd.	Range[min~max]
Raw Reads (million)	1353.64±128.25	[1128.91~1653.91]
Clean Reads (%)	87.73±2.39	[82.35~90.85]
Clean reads Q20 (%)	99.15±0.16	[98.79~99.38]
Map rate (%)	98.01±0.38	[96.93~98.65]
Mean depth (×)	37.65±4.31	[29.29~47.35]
Coverage at 1 × depth (%)	94.62±0.3	[94.15~94.89]
Coverage at 2 × depth (%)	94.51±0.32	[93.98~94.81]
Coverage at 4 × depth (%)	94.31±0.34	[93.69~94.67]
Coverage at 10 × depth (%)	93.56±0.38	[92.72~94.12]

Supplementary Table S2. 114 rare genes related to ALS from databases and papers

Gene symbol	Gene name	Classification	Known inheritance pattern	pHaplo	pTriplo
ALS2	alsin Rho guanine nucleotide exchange factor ALS2	causative gene	AR	0.8	0.52
ANG	angiogenin	causative gene	AD*	0.15	0.19
ANXA11	annexin A11	causative gene	AD	0.21	0.27
C9orf72	C9orf72-SMCR8 complex subunit	causative gene	AD	0.37	0.15
CCNF	cyclin F	causative gene	AD	0.77	0.36
CHCHD10	coiled-coil-helix-coiled-coil-helix domain containing 10	causative gene	AD	0.2	0.19
CHMP2B	charged multivesicular body protein 2B	causative gene	AD	0.11	0.22
ERBB4	erb-b2 receptor tyrosine kinase 4	causative gene	AD	**0.97**	0.79
FIG4	FIG4 phosphoinositide 5-phosphatase	causative gene	AD	0.24	0.32
FUS	FUS RNA binding protein	causative gene	AD	**0.9**	**0.94**
HNRNPA1	heterogeneous nuclear ribonucleoprotein A1	causative gene	AD	**0.92**	0.88
MATR3	matrin 3	causative gene	AD	**0.97**	**0.99**
OPTN	optineurin	causative gene	AD, AR	0.38	0.6
PFN1	profilin 1	causative gene	AD	**0.95**	0.7
SETX	senataxin	causative gene	AD	**0.97**	0.88
SIGMAR1	sigma non-opioid intracellular receptor 1	causative gene	AR	0.22	0.62
SOD1	superoxide dismutase 1	causative gene	AD, AR	0.75	**0.68**
SPG11	SPG11 vesicle trafficking associated, spatacsin	causative gene	AR	0.21	0.32
SQSTM1	sequestosome 1	causative gene	AD	0.51	**0.97**
TARDBP	TAR DNA binding protein	causative gene	AD	**0.97**	0.91
TBK1	TANK binding kinase 1	causative gene	AD	0.49	0.8
TIA1	TIA1 cytotoxic granule associated RNA binding protein	causative gene	AD	0.71	0.85
TUBA4A	tubulin alpha 4a	causative gene	AD	0.47	0.86
UBQLN2	ubiquilin 2	causative gene	XLD	NA	NA
VCP	valosin containing protein	causative gene	AD	**0.93**	**1**
CYLD	*CYLD* lysine 63 deubiquitinase	causative gene	AD	**0.99**	**0.98**
SPTLC1	serine palmitoyltransferase long chain base subunit 1	causative gene	AD	0.27	0.11
WDR7	WD repeat domain 7	causative gene	unknown	**0.94**	0.59
CFAP410	cilia and flagella associated protein 410	causative gene	AD	NA	NA
ALS3	-	loci associated with ALS	AD	NA	NA
ALS7	-	loci associated with ALS	unknown	NA	NA

PPARGC1A	PPARG coactivator 1 alpha	MGI-associated gene	unknown	**0.96**	0.8
TFAM	transcription factor A, mitochondrial	MGI-associated gene	unknown	0.53	0.4
TXNRD1	thioredoxin reductase 1	MGI-associated gene	unknown	0.59	0.64
ADARB1	adenosine deaminase RNA specific B1	MGI-associated gene	AR	**0.89**	0.9
PSMC4	proteasome 26S subunit, ATPase 4	MGI-associated gene	unknown	0.43	**1**
ZNF106	zinc finger protein 106	MGI-associated gene	unknown	0.56	0.68
EPG5	ectopic P-granules 5 autophagy tethering factor	MGI-associated gene	AR	0.65	0.39
ATXN2	ataxin 2	susceptibility to ALS	AD	**0.98**	**0.96**
DCTN1	dynactin subunit 1	susceptibility to ALS	AD, AR	0.36	0.93
KIF5A	kinesin family member 5A	susceptibility to ALS	AD	0.49	0.92
NEFH	neurofilament heavy	susceptibility to ALS	AD, AR	0.29	0.51
NEK1	NIMA related kinase 1	susceptibility to ALS	AD	0.81	0.48
PRPH	peripherin	susceptibility to ALS	AD, AR	0.15	0.06
GRB14	growth factor receptor bound protein 14	susceptibility to ALS	unknown	0.44	0.34
MAPT	microtubule associated protein tau	susceptibility to ALS	AR	0.3	0.18
SOD2	superoxide dismutase 2	susceptibility to ALS	unknown	0.58	0.33
TRPM7	transient receptor potential cation channel subfamily M member 7	susceptibility to ALS	AD	**0.9**	0.24
CNTN6	contactin 6	Modify gene	unknown	0.15	0.05
UNC13A	unc-13 homolog A	Modify gene	AD	0.76	**0.95**
CX3CR1	C-X3-C motif chemokine receptor 1	Modify gene (lifetime and disease progress)	unknown	0.1	0.16
CAMTA1	Calmodulin brinding transcriptions activator 1	Modify gene (lifetime)	AD	**1**	**1**
PON1	paraoxonase 1	Modify gene (lifetime)	unknown	0.25	0.31
SPAST	spastin	Modify gene (lifetime)	AD	**0.98**	0.81
ENAH	ENAH actin regulator	Modify gene (disease development)	AR	**0.98**	**0.96**
OGG1	8-oxoguanine DNA glycosylase	Modify (respiratory system)	AR,AD	0.59	0.55
PNPLA6	patatin like phospholipase domain containing 6	associated-*FUS*ion gene	unknowm	0.24	0.84
DOC2B	double C2 domain beta	associated-therapy	AR	0.82	0.22
LOX	lysyl oxidase	associated-therapy	AR	**0.89**	0.80
VEGFA	vascular endothelial growth factor A	associated-therapy	unknown	0.58	0.45
CNTN4	contactin 4	Strong evidence	AR	0.7	0.18
CRIM1	cysteine rich transmembrane BMP regulator 1	Strong evidence	AR,AD	**0.98**	0.58
CYP2D6	Cytochrome P450 family 2 subfamily D member 6	Strong evidence	unknown	0.38	0.35
KDR	kinase insert domain receptor	Strong evidence	unknown	**0.89**	**0.99**

AR	androgen receptor	Moderate evidence	AR,AD	0.09	0.28
ARHGEF28	rho guanine nucleotide exchange factor 28	Moderate evidence	unknown	0.72	0.82
ARPP21	cAMP Regulated Phosphoprotein 21	Moderate evidence	unknown	0.27	0.1
ATXN1	ataxin 1	Moderate evidence	AD	**0.99**	**0.94**
CCS	copper chaperone for superoxide dismutase	Moderate evidence	unknown	0.12	0.62
CDH13	cadherin 13	Moderate evidence	AD	0.84	0.25
CDH22	cadherin 22	Moderate evidence	unknown	0.79	0.81
APEX1	apurinic/apyrimidinic endodeoxyribonuclease 1	Tenuous (ALSoD)	unknown	0.51	0.56
DISC1	DISC1 scaffold protein	Tenuous (ALSoD)	XLR	0.77	0.43
DPP6	dipeptidyl peptidase like 6	Tenuous (ALSoD)	unknown	0.82	0.12
DYNC1H1	dynein cytoplasmic 1 heavy chain 1	Tenuous (ALSoD)	unknown	**0.98**	**1**
EFEMP1	EGF containing fibulin extracellular matrix protein 1	Tenuous (ALSoD)	AD	0.81	0.55
EWSR1	EWS RNA binding protein 1	Tenuous (ALSoD)	AD	**0.99**	**0.99**
FEZF2	FEZ family zinc finger 2	Tenuous (ALSoD)	unknown	**0.97**	**0.97**
GLT8D1	glycosyltransferase 8 domain containing 1	Tenuous (ALSoD)	AR	0.25	0.43
GRN	granulin precursor	Tenuous (ALSoD)	AD	0.16	0.34
HEXA	hexosaminidase subunit alpha	Tenuous (ALSoD)	AD	0.44	0.5
HFE	homeostatic iron regulator	Tenuous (ALSoD)	AD	0.21	0.16
ITPR2	inositol 1,4,5-triphosphate receptor type 2	Tenuous (ALSoD)	unknown	0.85	0.73
KIFAP3	kinesin associated protein 3	Tenuous (ALSoD)	unknown	0.34	0.54
LMNB1	lamin B1	Tenuous (ALSoD)	AD	0.74	0.29
NEFL	neurofilament light	Tenuous (ALSoD)	AD	NA	NA
ODR4	odr-4 GPCR localization factor homolog	Tenuous (ALSoD)	AD	NA	NA
PLEKHG5	pleckstrin homology and RhoGEF domain containing G5	Tenuous (ALSoD)	AR,AD	0.71	0.63
PON2	paraoxonase 2	Tenuous (ALSoD)	unknown	0.32	0.33
RAMP3	receptor activity modifying protein 3	Tenuous (ALSoD)	AR	0.19	0.24
SARM1	sterile alpha and TIR motif containing 1	Tenuous (ALSoD)	AR	0.17	0.81
SCN7A	sodium voltage-gated channel alpha subunit 7	Tenuous (ALSoD)	unknown	0.69	0.32
SEMA6A	semaphorin 6A	Tenuous (ALSoD)	unknown	**0.98**	**0.94**
SLC39A11	solute carrier family 39 member 11	Tenuous (ALSoD)	unknown	0.5	0.15
SLC52A3	solute carrier family 52 member 3	Tenuous (ALSoD)	unknown	0.43	0.1
SPG7	SPG7 matrix AAA peptidase subunit, paraplegin	Tenuous (ALSoD)	AR	0.52	0.18
SS18L1	SS18L1 subunit of BAF chromatin remodeling complex	Tenuous (ALSoD)	unknown	0.82	0.9

SUSD1	sushi domain containing 1	Tenuous (ALSoD)	AR,AD	0.17	0.37
SYNE1	spectrin repeat containing nuclear envelope protein 1	Tenuous (ALSoD)	unknown	0.87	**1**
TAF15	TATA-box binding protein associated factor 15	Tenuous (ALSoD)	AR,AD	0.23	0.69
TNIP1	TNFAIP3 interacting protein 1	Tenuous (ALSoD)	unknown	0.71	0.48
VRK1	VRK serine/threonine kinase 1	Tenuous (ALSoD)	AR	0.75	0.56
ZFP64	ZFP64 zinc finger protein	Tenuous (ALSoD)	unknown	**0.88**	0.4
ZNF512B	zinc finger protein 512B	Tenuous (ALSoD)	unknown	**0.99**	**0.96**
AGT	angiotensinogen	Tenuous (ALSoD)	AR	0.43	0.16
DIAPH3	diaphanous related formin 3	conflicting	unknown	0.32	0.15
DNAJC7	DnaJ heat shock protein family (Hsp40) member C7	conflicting	unknown-conflicting	0.8	**1**
NIPA1	NIPA magnesium transporter 1	conflicting	unknown	0.11	0.53
RBCK1	RANBP2-type and C3HC4-type zinc finger containing 1	weakness evidence	AR	**0.94**	0.53
TRIB3	tribbles pseudokinase 3	weakness evidence	unknown	0.63	0.11
SNPH	syntaphilin	weakness evidence	unknown	**0.93**	0.45
CDC25B	cell division cycle 25B	weakness evidence	unknown	0.78	**1**
TGM6	transglutaminase 6	weakness evidence	AD	NA	NA

*Gene symbol and Gene name were checked on the web of HGNC (http://www.genenames.org). AD: autosomal dominant. AR: autosomal recessive. XLR: chromosome X linked recessive. pHaplo: Predicted Probability of Haploinsufficiency, pTriplo: Predicted Probability of Triplosensitivity (http://www.deciphergenomics.org).
